# Substructural Connectivity Fingerprint and Extreme Entropy Machines—A New Method of Compound Representation and Analysis

**DOI:** 10.3390/molecules23061242

**Published:** 2018-05-23

**Authors:** Krzysztof Rataj, Wojciech Czarnecki, Sabina Podlewska, Agnieszka Pocha, Andrzej J. Bojarski

**Affiliations:** 1 Institute of Pharmacology, Polish Academy of Sciences, Department of Medicinal Chemistry, Smętna Street 12, 31-343 Kraków, Poland; rataj@if-pan.krakow.pl (K.R.); smusz@if-pan.krakow.pl (S.P.); 2 Faculty of Mathematics and Computer Science, Jagiellonian University, Łojasiewicza Street 6, 30-348 Kraków, Poland; lejlot@gmail.com (W.C.); agnieszka.pocha@doctoral.uj.edu.pl (A.P.)

**Keywords:** fingerprint, molecular representation, machine learning, substructures

## Abstract

Key-based substructural fingerprints are an important element of computer-aided drug design techniques. The usefulness of the fingerprints in filtering compound databases is invaluable, as they allow for the quick rejection of molecules with a low probability of being active. However, this method is flawed, as it does not consider the connections between substructures. After changing the connections between particular chemical moieties, the fingerprint representation of the compound remains the same, which leads to difficulties in distinguishing between active and inactive compounds. In this study, we present a new method of compound representation—substructural connectivity fingerprints (SCFP), providing information not only about the presence of particular substructures in the molecule but also additional data on substructure connections. Such representation was analyzed by the recently developed methodology—extreme entropy machines (EEM). The SCFP can be a valuable addition to virtual screening tools, as it represents compound structure with greater detail and more specificity, allowing for more accurate classification.

## 1. Introduction

Modern drug discovery calls for more cost-efficient and effective methods of filtering the vast libraries of chemical compounds in the search for potential drugs. Since in vitro screening is rather expensive and time-consuming, the attention of the researchers turns towards the in silico methods more than ever before. The numerical methods of compound screening and selection, collectively called virtual screening (VS) [1], play a major role in the process of computer-aided drug design (CADD) [2].

The key-based substructural fingerprints (FPs) [3] are a popular method of compound representation used in early stages of a VS cascade. They are based on the occurrences of predefined chemical groups—“keys”, and are encoded as a bit string that can be easily analyzed using various algorithms, such as similarity searching, hierarchical clustering or activity-based discrimination tests using machine learning (ML) methods. There are several available key-based FPs, differing in the set of keys used for their generation, e.g., Klekota–Roth FP (KR) [4], MACCS FP [5], Substructure FP (SUB) [6], or CACTVS FP (or PubChem FP) [7].

Fingerprint-based filtering is very often used in the VS cascade, due to its relative simplicity. This ligand-based methodology is computationally cheap, compared with structure-based approaches. Moreover, it requires a very limited amount of data and, what must also be considered, the algorithms used for fingerprint generation and analysis are usually readily available and free to use. The initial processing of the compound libraries with the key-based substructural FPs may greatly reduce the number of compounds used in further steps by discarding the compounds with low-to-none probability of being active towards the considered biological target.

**Figure 1:**
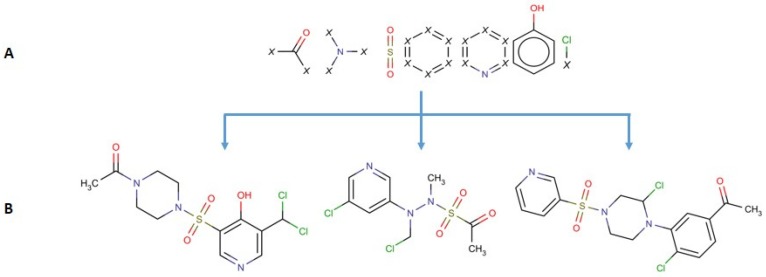
For the seven depicted substructures: (**A**) all three compounds (**B**) share identical fingerprint, despite major structural differences.

Despite their undeniable usefulness, the key-based methods possess a major flaw: the relative positions of the chemical groups are neglected during the FP calculation. This may lead to cases where two significantly different compounds may share a very similar or even identical fingerprint due to the possession of the same chemical groups in their structures (although differently connected) (Figure 1).

**Figure 2:**
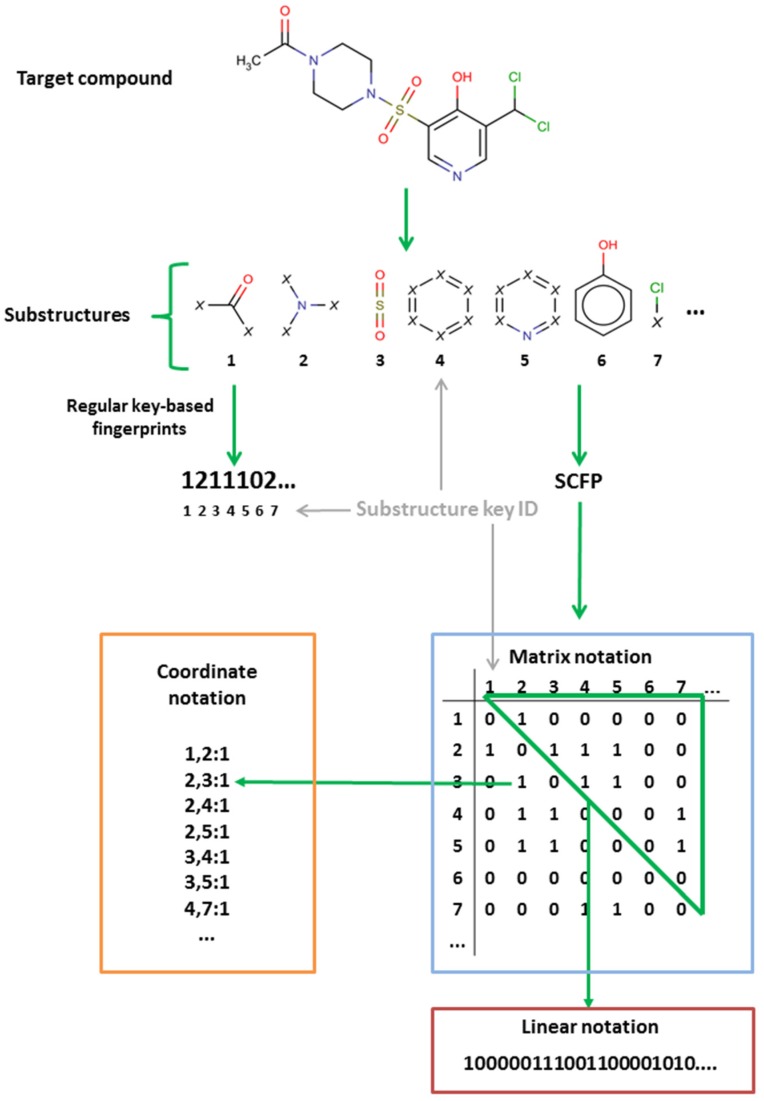
Comparison of standard key-based substructural FPs and SCFP.

In this study, we strive to improve the key-based substructural FPs by introducing connectivity data, thus creating a new method of compound representation—the substructural connectivity fingerprint (SCFP). The form of SCFP is a matrix of occurrences and connections between substructural features (Figure 2), which leads to a much more consistent representation than standard key-based FPs.

Despite its more complex nature, the SCFP can be analyzed with standard methods, such as similarity metrics, regular ML algorithms, support vector machines (SVM) [8], as well as more sophisticated graph-based algorithms or extreme entropy machines (EEM) [9]. The classification abilities of the new representation method were tested on sets of compounds for targets previously researched in our lab possessing relatively balanced compound sets, that is G-protein coupled receptors (GPCR) [10] and the serotonin transporter (SERT) [11]. We used multiple classification algorithms: SVM with two various kernels (Radial Basis Function, RBFSVM and Tanimoto, TanSVM), the EEM methodology and Naïve Bayes (NB). Classification tests were run as five-fold cross-validation and the metric used for their evaluation was balanced accuracy (BAC). The SCFP was generated based on three substructure key sets: KR, SUB and MACCS and was tested against regular key-based substructural FPs.

## 2. Results and Discussion

In this study, the classification efficiency of the SCFP was tested on compound sets for 11 target proteins (using active and inactive compounds extracted from ChEMBL database), using 5 ML analysis methods and 3 various substructure key sets (Table 1).

**Figure 3:**
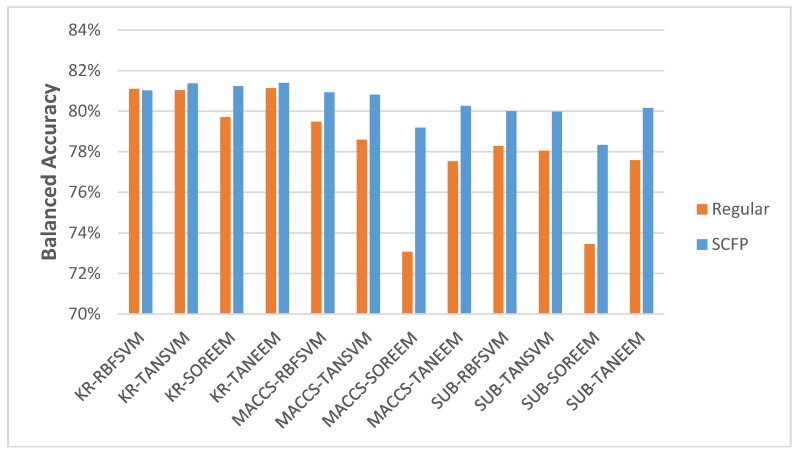
The average BAC scores achieved by five-fold cross-validation discrimination tests using various key sets: MACCS, Substructure (SUB) and Klekota-Roth (KR), as well as SVM and EEM methods employing various kernels: Radial Basis Function (RBF), Tanimoto (TAN) and Sorensen (SOR).

Most importantly, the results demonstrate that the SCFP outperformed their one-dimensional equivalents. The SCFP analyzed with all ML methods indicates an increase in classification accuracy, which is especially visible when applied to MACCS and SUB FPs (Figure 3). The standard errors of classification using SCFP remain comparable in value to those when using standard key-based FPs (data in Supplementary Material).

**Figure 4:**
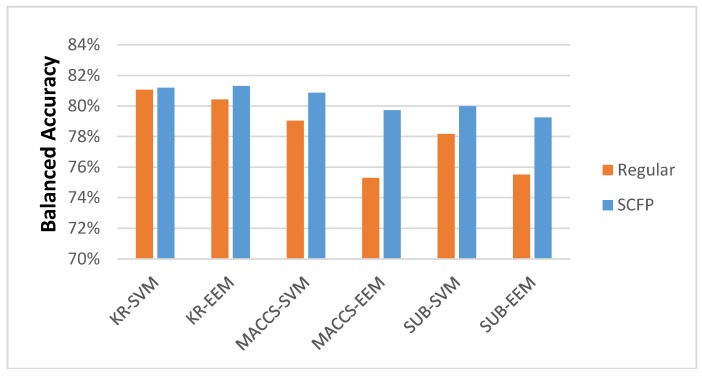
Average BAC scores achieved by five-fold cross-validation classification tests when the best kernel for each classification test was considered.

We can see that the increase in accuracy is significant, when compared to regular key-based FPs. When we disregard the kernels used for classification, the data show a slight increase in accuracy (Figure 4).

**Figure 5:**
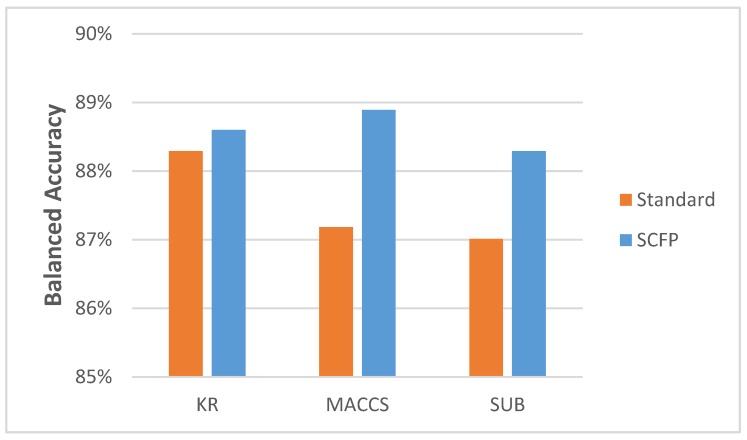
Average BAC scores achieved in five-fold cross-validation classification tests when the best results are taken into consideration, regardless of ML method or kernel used.

However, if we employ the approach of running multiple ML methods and kernels and choose only the combination with the best results, the SCFP is becoming a much better representation (Figure 5).

The classification tests were also performed on targets with highly unbalanced ligand sets, in order to verify the stability of the methodology. The chosen proteins were protein kinases, which were also used in our previous research [12] (Table 2).

**Figure 6:**
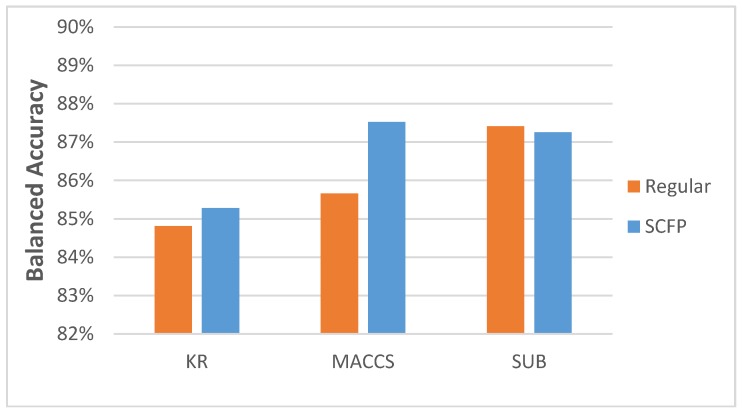
Average BAC scores achieved in five-fold cross-validation classification tests for highly unbalanced compound sets.

We can observe, that the SCFPs based on KR keys fare much worse, when faced with highly unbalanced sets (Figure 6). In such case, the MACCS-based SCFP achieves highest results, while SUB-based SCFP experienced a drop in classification accuracy.

In almost all presented cases one can observe better performance of the SCFP over the standard key-based FP. These results indicate that the implementation of SCFP into VS campaigns has the potential to significantly improve the quality of initial filtering of compound databases, which in turn would lead to both reduction in time and cost as well as an increase of efficacy of further research.

Keeping in mind that the SCFP is fully interpretable, i.e., each bit can be translated to a certain pair of substructures, this fingerprint allows for more sophisticated studies on the structure of chemical compounds.

What is more, the EEM in many cases fares better than the state-of-the-art SVM methodology. It is an interesting observation, that once the compounds are represented with SCFP, the EEM predictive power becomes much stronger compared to regular FPs. This result not only shows that the FP itself is a significant addition to the existing representations, but also the connection of EEM and the SCFP is a major improvement for the field. While the time cost of SCFP generation is slightly higher, the analysis of larger sets of compounds using EEM becomes relatively faster to other ML methods.

This study shows that additional information conveyed by a FP can be successfully used in VS and that there is room for improvement in the current state-of-the-art methodology. The SCFP offers advantages in VS campaigns, and has the power to improve screening results.

## 3. Materials and Methods

In this study, we chose six serotonin receptors (5-HT_n_R; n: 1A, 1B, 2A, 2B, 6, 7) and four other GPCRs: beta2 adrenergic receptor (beta2-AR), histamine receptor type 1 (H1), muscarine receptor type 2 (M2), metabotropic glutamate receptor type 3 (mGluR3) and the SERT. These proteins were chosen because various FP-based analyses for those targets were previously conducted in our lab [10,11].

Additionally, five protein kinases were selected for research on unbalanced compound sets: the tyrosine protein kinase ABL, the cyclin-dependent kinase 2 (CDK2), the glycogen synthase kinase-3 beta (GLY), the tyrosine protein kinase LCK, and the tyrosine protein kinase SRC, also analyzed in previous research [12]. To perform the classification, sets of compounds with known activity towards the target proteins were acquired. The compounds were extracted from the ChEMBL20 [13] database and were divided into two groups based on their activity described by the inhibition constant (K_i_) (or equivalent, i.e., pK_i_, IC_50_, and logIC_50_, assuming that K_i_ = IC_50_/2 [14]: actives (K_i_ < 100 nM) and inactives (K_i_ > 1000 nM) (Table 1). The compound sets are available in the Supplementary Material.

In the study we used three substructure key sets: KR, SUB and MACCS (MACCS 166). In result, for each target and each compound group regular Substructure, MACCS and KR FPs were calculated using PaDEL-Descriptor [15] software, as well as the SCFP representation that used those substructure keys definitions extracted from the PaDEL parameters. In this research, the efficiency of the SCFP method was analyzed by classification experiments using EEM, SVM, and NB kernels.

### 3.1. SCFP Generation

**Figure 7:**
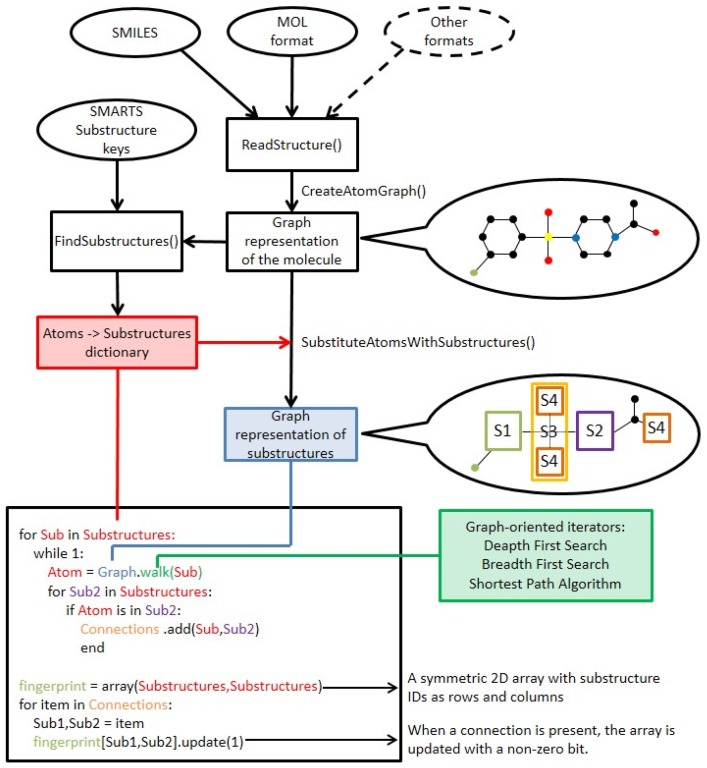
The graphic representation of the SCFP generation algorithm.

**Figure 8:**
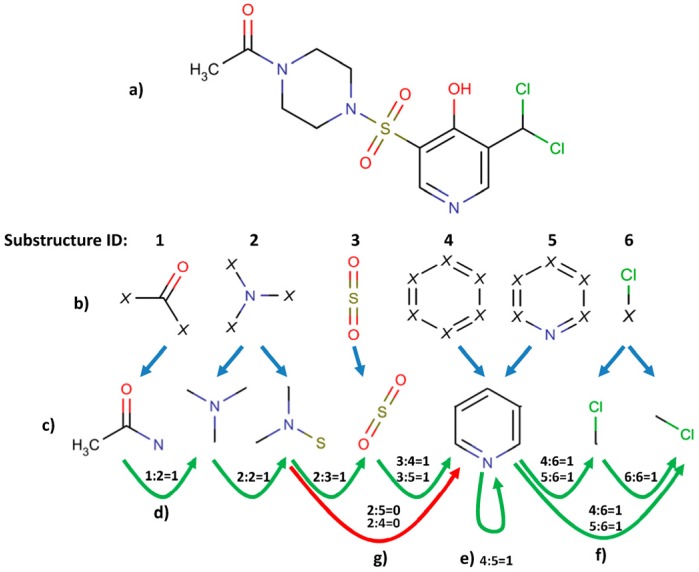
Detailed representation of SCFP calculation. The compound is imported (**a**) and it is screened for predefined substructures; (**b**) all the found substructures are extracted; (**c**) and connections between them are determined. If there is no other predefined substructure present between two substructures, they are regarded as being connected; (**d**) bit value set to 1; sharing atoms between substructures also counts (**e**). The number of such connections is not recorded, therefore one substructure connecting to two substructures of the same kind is treated exactly the same as connection to one substructure; (**f**) if there is any predefined substructure present between two substructures, they are considered as not connected; (**g**) bit set to 0.

The SCFP is an extension to existing key-based substructural FPs, and are based on similar principles. The methodology of SCFP construction is as follows (Figure 7 and Figure 8):

1. The molecule is read from SDF, MOL or SMILES format and built into a graph representation, where the nodes represent atoms and edges represent chemical bonds.
2. Substructure keys in SMARTS format are loaded.
3. A two-dimensional symmetric array is created, where substructure key IDs are represented as column and row headers.
4. A standard substructure search is conducted using the substructure keys. Each hit is recorded, and the atoms that comprise the substructure are saved.
5. The molecular graph is substituted with a substructure graph, where nodes are substructures and edges are connections between them. In case an atom did not belong to any of the substructures, it is represented as a node as well.
6. For each atom of every hit, a walking algorithm is used to find the nearest other hit in the molecule graph.
7. If a hit is found, the event is stored within the SCFP matrix (Substructure_1:Substructure_2 = 1).
8. The fingerprint may be recorded in multiple ways, i.e., matrix, coordinate and linear notation.

### 3.2. Classification

The classification procedure was performed using five-fold cross-validation approach with known active (positive data points) and inactive (negative data points) compounds extracted from the ChEMBL database. The five-fold cross-validation method entails dividing both the positive and negative data points into five even randomly selected subsets. Out of those, four positive and four negative subsets are merged to form the training set, which is the used to teach the algorithm to discriminate between positive and negative data. The remaining subsets of both positive and negative data is then used as an evaluation set, where the algorithm tries to classify the new examples. Since the actual class of the evaluation data is known, it is possible to calculate the efficacy of the method. This process is repeated five times, so that every subset (out of the original five) is used as the evaluation data. Due to imbalance of positive/negative samples in analyzed datasets we use BAC as an evaluation metric:

(1) $$\mathrm{BAC}\left(\mathrm{TP},\mathrm{TN},\mathrm{FP},\mathrm{FN}\right)=\;\frac{1}{2}\left(\frac{\mathrm{TP}}{\mathrm{TP}+\mathrm{FN}}+\frac{\mathrm{TN}}{\mathrm{TN}+\mathrm{FP}}\right)$$

where: TP—True Positive, FP—False Positive, TN—True Negative, FN—False Negative.

The hyperparameter selection for the ML kernels was conducted: for SVM with RBF kernel we determined optimal C (10^−3^–10^6^, step of power 1) and γ (10^−3^–10^5^, step of power 1; RBF kernel width), for SVM with Tanimoto kernel we selected C (values as in RBF kernel), for EEM we selected h (0.1, 0.2, 0.5, 1.0) and C (0, 100, 1000, 100,000), and for Naïve Bayes α—smoothing factor for probability estimators (0.01, 0.1, 0.2, 0.5, 1.0).

For each of the targets and each of the basic substructural fingerprints, a total of 117 classifiers were built (kernels with various hyperparameters, selected in a grid-like manner), out of which only the one with the best BAC value was considered for comparison. This procedure was conducted for both SCFP and original fingerprints.

### 3.3. Implementation

The basic SCFP creating algorithm uses Schrodinger’s Canvas suite [16] for reading molecules and substructure searching. The entirety of code is written in Python.

For ML algorithms, we used code also written in Python, with the use of scikit-learn, numpy and scipy. For SVM we use the LIBSVM library through the scikit-learn SVC class, for Random Forest and Naïve Bayes implementations from the same library were applied. Only EEM was implemented from scratch in Python so its training time is overestimated (it can be much faster using low-level code such as the competitive methods).

## 4. Conclusions

In this research we presented the SCFP as a new method of compound representation. SCFP merges the advantages of standard key-based substructural and reduced graphs methods, leading to an increase in compound classification efficiency. The addition of intra-substructural connectivity data into the FP allows for the acquisition of more specific substructure patterns within compounds, which in turn enables classification algorithms to more accurately filter out inactive compounds that structurally resemble active ones. The SCFP uses substructural keys from other FPs, and therefore different results can be achieved with different keys used. However from the three sets available in PaDEL software (MACCS, SUB, KR), the MACCS-based and KR-based SCFP gave the best results. Although the KR-based SCFP presented the smallest improvement, its original efficiency still renders it as one of the best FP for discrimination tests. We believe that the small increase in efficiency is caused by the nature of substructure keys present in the KR FP: the keys are strongly defined, leaving little room for substructure divergence. This mostly results in cases, where the connection of two substructures is synonymous with the existence of a single, larger substructure within the fingerprint. The biggest increase in the BAC value was in case of the MACCS keys, where each key is much less unequivocal than the ones seen in the KR FP. It is worth noticing that the SCFP built with MACCS keys performed similarly to the SCFP built with KR keys, which is a significant achievement. Although the accuracy increase is relatively small, the nature of the this representation, together with its efficiency, opens up a new spectrum of possible uses, further enhancing the VS procedures. For example, since compounds active towards proteins that are closely related are highly similar, the SCFP may enable searching for selective compounds based on the small differences and not captured by regular FPs. Nevertheless, it is also possible to customize the substructure keys used by SCFP, which should enable more target-specific classification.

The SCFP itself is greatly beneficial; however, when analyzed with the EEM, it grants an additional boost to accuracy, compared with kernelized SVM or NB.

To summarize the major points from this study:

1. The proposed SCFP provides an easy to compute, alternative representation of chemical compounds.
2. SCFP can be easily incorporated into the existing modern ML models such as kernelized SVM or EEM.
3. The SCFP significantly increases the quality of trained models.
4. The combination of SCFP with EEM further increases the accuracy while preserving simplicity and speed.

The increase in accuracy is significant, even though very simple version of the fingerprint was used. Future plans concerning the SCFP methodology include adding more types of data, such as defining types of connections (e.g., direct, indirect, atom-sharing etc.).

The SCFP generation algorithm as well as machine learning implementations are available on demand or at medchem-ippas.eu.

## Figures and Tables

**Table 1:** Number of compounds in used datasets.

Protein	Number of Actives	Number of Inactives
5-HT1A	4166	1155
5-HT1B	628	297
5-HT2A	1870	976
5-HT2B	407	333
5-HT6	1490	341
5-HT7	702	370
Beta2-AR	275	350
H1	659	558
M2	288	261
mGluR3	48	86
SERT	3692	1596

**Table 2:** Number of compounds for protein kinases in used datasets.

Protein	Number of Actives	Number of Inactives
ABL	4166	1155
CDK2	628	297
GLY	1870	976
LCK	407	333
SRC	1490	341

## Supplementary Materials

Supplementary materials can be found at http://www.mdpi.com/1420-3049/23/6/1242/s1. S1 Xls File: the spreadsheet with all the data acquired from the machine learning tests, divided into tabs for each substructural key set used. S2 Tar file: the compound sets used in the study, delivered in SDF format, divided into actives and inactives for each target protein.

## Abbreviations

VS	Virtual screening
CADD	Computer-aided drug design
FP	Fingerprint
SUB	Substructural fingerprint
ML	Machine learning
SCFP	Substructural connectivity fingerprints
SVM	Support vector machines
EEM	Extreme entropy machines
GPCR	G protein-coupled receptor
SERT	Serotonin transporter
BAC	Balanced accuracy
RBFSVM	Support vector machines with radial basis function kernel
TanSVM	Support vector machines with Tanimoto kernel
NB	Naïve Bayes
Beta2-AR	Beta 2 adrenergic receptor
H1	Histamine receptor type 1
M2	Muscarinic receptor type 2

## Appendix A. Machine Learning

In this section we briefly summarize the methods used in this study. We denote a training set in the form of binary labelled samples, where $x_{i}$ is a compound representation in ${\mathbb{R}}^{d}$, and $t_{i}\in \left\{-1,+1\right\}$ denotes the compound’s activity.

### Appendix A.1. Balanced Naïve Bayes

NB is the simplest probabilistic classifier which assumes that features of ith compound $x_{i}^{j}$ (elements of the fingerprint) are conditionally independent given the compound activity $t_{i}$. To maximize the accuracy of the model, the posterior probability p(t|x) can be computed using:

$$p\left(t|x\right)=\frac{\prod_{j=1}^{d}p\left(x^{j}|t\right)p\left(t\right)}{p\left(x\right),}$$

where $p\left(x^{j}|t\right)$ is a conditional probability estimated by counting the occurrences of a particular feature in each class, p(t) is a class prior estimated as the ratio of the particular class samples to the total number of training points, and p(x) is a per-sample probability that can be omitted during classification.

If one wishes to maximize a different evaluation metric such as balanced accuracy, fixed priors p(t) instead of empirically estimated ones. For BAC, we simply apply p(t=−1)=p(t=+1)=1/2, which leads to a very simple classification rule:

$$\mathrm{cl}\left(x\right)=\underset{t\in \left\{-1,+1\right\}}{\arg\;\max}\frac{\prod_{j=1}^{d}p\left(x^{j}|t\right)p\left(t\right)}{p\left(x\right)}=\underset{t\in \left\{-1,+1\right\}}{\arg\;\max}\overset{d}{\underset{j=1}{\prod }}\left(x^{j}|t\right).$$

### Appendix A.2. Balanced Support Vector Machines

A SVM tries to separate data points using a hyperplane with normal β and maximizing the margin—the distance between the closest points of each class. This process can be formally stated by:

$$\underset{\beta ,b}{\mathrm{minimize}}\frac{1}{2}{\left\|\beta \right\|}^{2}+C\overset{N}{\underset{i}{\sum }}\xi_{i}\;\mathrm{subject}\;\mathrm{to}\;t_{i}\left(\langle \beta ,x_{i}\rangle -b\right)\geq 1-\xi_{i},\;i=1,\ldots ,N,$$

where C denotes the trade-off between a correct fitting to the data (high value) and the strength of the regularization (simplicity of the model, small value).

The above problem introduced by Vapnik [8] can be further generalized into a nonlinear model by exploitation of the custom kernel function K:

$$\underset{\lambda }{\mathrm{maximize}}\overset{N}{\underset{i=1}{\sum }}\lambda_{i}-\frac{1}{2}\overset{N}{\underset{1,j=1}{\sum }}\lambda_{i}\lambda_{j}t_{i}t_{j}K\left(x_{i},x_{j}\right)$$

$$\mathrm{subject}\;\mathrm{to}\;\overset{N}{\underset{i=1}{\sum }}\lambda_{i}t_{i}=0$$

$$0\leq \lambda_{i}\leq C,\;i=1,\ldots ,N.$$

The selection of a particular K function is known to be a crucial element for maximizing the model efficiency. It is worth noting that only a very small set of all possible similarity functions conform valid kernels, as the function needs to denote a correct dot product in some Hilbert space.

Furthermore, SVM maximizes the accuracy metric in this setting, which is unsuitable for highly unbalanced data sets (i.e., those containing far more inactive than active compounds) [1,17]. In order to balance the model to avoid focusing purely on the majority class, one must introduce sample weights that are inversely proportional to their class size:

$$\underset{\lambda }{\mathrm{maximize}}\overset{N}{\underset{i=1}{\sum }}\lambda_{i}-\frac{1}{2}\overset{N}{\underset{1,j=1}{\sum }}\lambda_{i}\lambda_{j}t_{i}t_{j}K\left(x_{i},x_{j}\right)$$

$$\mathrm{subject}\;\mathrm{to}\;\overset{N}{\underset{i=1}{\sum }}\lambda_{i}t_{i}=0$$

$$0\leq \lambda_{i}\leq C\cdot \frac{\max\left\{N_{-1},N_{+1}\right\}}{N_{t_{i}}},\;i=1,\ldots ,N,$$

where $N_{t}$ is the number of compounds for label t. As a result, samples from the majority class have smaller penalties and those from the minority class have larger penalties for misclassification.

While SVMs work well for data sets with a few thousand examples, the approach scales poorly when applied to larger problems. Furthermore, the kernel function, measuring the similarity of two samples, has to meet certain mathematical conditions in order for SVM to work. Recently, additional techniques have emerged that may help overcome these issues. In particular, EEM, proposed by Czarnecki et al. [9] shows a different approach, where one uses a family of random, nonlinear projections which transform data to a new space, in which the linear separation can be easily found

### Appendix A.3. Extreme Entropy Machines

Given a random transformation φ and constant C with similar interpretations to those in SVM, one can define the regularized version of EEM (as suggested by its authors [9])

$$\underset{\beta }{\mathrm{minimize}}\;\beta^{T}\left(\Sigma^{+}+\Sigma^{-}+\frac{1}{C}I\right)\beta$$

$$\mathrm{subject}\;\mathrm{to}\;\beta^{T}\left(m^{+}-m^{-}\right)=2$$

$${\mathrm{where}\;\Sigma }^{\pm }={\mathrm{cov}}_{†}\left(H^{\pm }\right)$$

$$m^{\pm }=\mathrm{mean}\left(H^{\pm }\right)$$

$$H^{\pm }=\varphi \left(X^{\pm }\right)$$

where ${\mathrm{cov}}_{†}$ is the Ledoit–Wolf covariance estimator [18].

The exact performance of EEM depends on the selection of φ, a random transformation of the input samples. One possibility is to use φ(x)=ϕ(x,w), where ϕ is some similarity measure, such as any kernel K. However, in contrast to SVM, *w* values are randomly selected from an arbitrary probability distribution, such as a Gaussian distribution. There are three very important differences between SVM and EEM:

1. The φ function of EEM can be an arbitrary projection, while K of SVM must meet the Mercer condition [19], which limits possible usage.
2. SVM optimization is a quadratic optimization problem with linear constraints and can be solved in $O\left(N^{3}\right)$ time using iterative solvers, where N is the number of training samples. The EEM problem can, in contrast, be solved directly (without iterative optimization) in $O\left(h^{2}N\right)$ where h is a tunable parameter (which is generally much smaller than N).
3. EEM is a balanced model, meaning that in its basic form it already maximizes the balanced accuracy measures; in contrast, SVM (and most of the other models) requires additional weighting of samples.
